# New persistent opioid use after colorectal surgery in Sweden: A nationwide register‐based cohort study

**DOI:** 10.1111/codi.70601

**Published:** 2026-08-24

**Authors:** Claes Gedda, Jonas Nygren, Erik Sturegård, Yunzhang Wang, Anders Thorell, Mattias Soop

**Affiliations:** ^1^ Department of Surgery and Anaesthesiology Ersta Hospital Stockholm Sweden; ^2^ Department of Clinical Science Danderyds Hospital, Karolinska Institute Stockholm Sweden; ^3^ Department of Translational Medicine, Faculty of Medicine Lund University Malmö Sweden; ^4^ Department of Communicable Disease Control and Preparedness Public Health Agency of Sweden Solna Sweden; ^5^ Department of Medical Epidemiology and Biostatistics Karolinska Institute Stockholm Sweden; ^6^ Department of Inflammatory Bowel Disease and Intestinal Failure Surgery Karolinska University Hospital Stockholm Sweden

**Keywords:** analgesics, colorectal surgery, opioid, persistent opioid use, postoperative pain, registries, Sweden

## Abstract

**Aim:**

To determine the incidence of new persistent opioid use (NPOU) after elective colorectal surgery in Sweden and to identify associated patient‐, procedure‐ and hospital‐level factors.

**Method:**

This nationwide retrospective cohort study linked data from Swedish national health registries and included adults undergoing elective inpatient colorectal surgery between 2018 and 2022. Patients with an opioid dispensed 365 to 30 days before surgery, another surgical procedure within one year of the index operation, or death within 12 months postoperatively were excluded. NPOU was defined as at least one opioid dispensed within 90 days after surgery and at least one between 90 and 180 days. Associations were examined using multivariable logistic regression.

**Results:**

Among 14,233 opioid‐naïve patients, 374 (2.63%; 95% CI, 2.37–2.90%) developed NPOU. Longer operating time (aOR, 1.19 per hour; 95% CI, 1.13–1.26), preoperative non‐opioid analgesics (aOR, 1.90; 95% CI, 1.52–2.37) or sedatives/anxiolytics (aOR, 1.66; 95% CI, 1.31–2.09), opioid dispensation within 7 days of surgery (aOR, 2.19; 95% CI, 1.76–2.72), and higher ASA classification (ASA 3: aOR, 1.89; 95% CI, 1.26–2.90; ASA 4: aOR, 3.29; 95% CI, 1.63–6.36) were independently associated with higher odds of NPOU, whereas male sex was associated with lower odds (aOR, 0.76; 95% CI, 0.61–0.95).

**Conclusion:**

In this low‐prescribing setting, approximately one in 38 opioid‐naïve patients developed NPOU 6 months after colorectal surgery. Operating time and opioid dispensation within 7 days of surgery were independently associated with NPOU; the latter, a modifiable prescribing decision, is a plausible target for opioid‐sparing strategies.

What does this paper add to the literature?

In a nationwide Swedish cohort of 14,233 opioid‐naïve patients undergoing elective colorectal surgery, 2.6% developed new persistent opioid use at 6 months follow‐up. Operating time and early opioid dispensation were independently associated with NPOU; operating time is a novel predictor in this setting, and early dispensing is a modifiable target for opioid‐sparing interventions.

## INTRODUCTION

New persistent opioid use (NPOU) is recognised as a common and serious adverse event following surgery [1]. The definition of NPOU has varied in previous studies. Recently, a definition focusing on continuous opioid use up to 6 months after surgery has been increasingly adopted [2, 3, 4, 5, 6]. As awareness of NPOU has increased, efforts have intensified to identify risk factors and patient groups at particular risk. Most research has focused on North America, although data are emerging from other parts of the world [3, 7]. Certain procedures, such as abdominal [8, 9] and orthopaedic spinal surgery [10, 11], appear to be associated with an increased incidence. However, even minor surgical procedures carry a measurable risk of new persistent opioid use. In a large US cohort study, Brummett et al. reported persistent opioid use in approximately 6% of opioid‐naïve patients after both major and minor operations [5].

Approximately 40% of all opioid overdose deaths involve prescription opioids, and 80% of patients diagnosed with an opioid use disorder have previously received an opioid prescription [12, 13].

Data from Sweden and the Nordic region remain scarce. The only previous Swedish population‐based study, by Lindeberg et al., reported a 7% incidence of NPOU across all major surgical specialities in a multicentre cohort from 2007 to 2014, identifying psychiatric comorbidity as the strongest independent predictor [14]. However, that study did not examine colorectal surgery specifically, did not include perioperative opioid dispensing or operating time as candidate predictors, and predates the widespread implementation of enhanced recovery after surgery (ERAS) protocols. Although postoperative opioid prescribing rates in Sweden are substantially lower than in North America, NPOU after colorectal surgery has not been systematically studied [15].

The Swedish healthcare system provides favourable conditions for high‐quality population research. Every resident is assigned a unique personal identity number, enabling precise linkage across nationwide registers that capture virtually all hospital admissions, outpatient visits, prescribed drugs and causes of death. This infrastructure allows for near‐complete longitudinal follow‐up without selection bias or loss to follow‐up.

We therefore aim to describe the incidence of NPOU 6 months after colorectal surgery in Sweden and to identify any associated perioperative factors. We hypothesised that the incidence of NPOU in Sweden would be lower than that reported in US cohorts.

## METHODS

This nationwide retrospective cohort study used data from multiple Swedish national health registries to identify all adult patients who underwent colorectal surgery in the five‐year period between January 1st, 2018, and December 31st, 2022. The publicly funded healthcare system in Sweden provides universal coverage, and the use of a unique personal identity number enables accurate linkage across nationwide registers. Data were extracted from four nationwide registers. The Swedish Perioperative Register (SPOR) records all surgical procedures, with 99% coverage; the National Prescribed Drug Register (NPDR) covers all dispensed prescriptions; the National Patient Register (NPR) includes all in‐ and outpatient hospital visits; and the Swedish Cause of Death Register (NCDR) records all deaths [16, 17]. Reporting to the national health registries is mandatory according to Swedish law [17].

**FIGURE 1:**
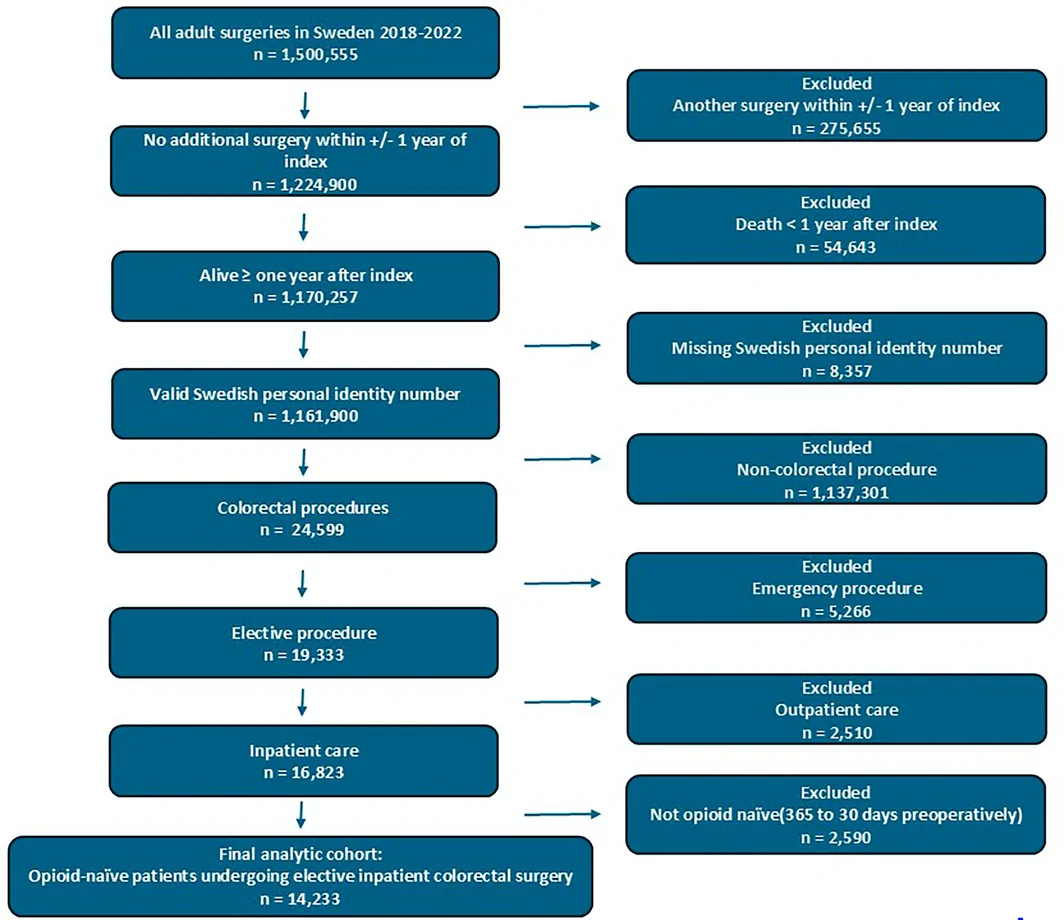
Flow diagram of cohort selection, from all adult operations recorded in the Swedish Perioperative Registry 2018–2022 to the final cohort of 14,233 opioid‐naïve patients undergoing elective inpatient colorectal surgery. Exclusions were applied sequentially in the order shown. KVÅ, Swedish Classification of Health Interventions; N02A, Anatomical Therapeutic Chemical code for opioid analgesics.

SPOR was used to identify the study cohort, comprising all patients aged 18 years or older who underwent elective, inpatient colorectal surgery. Procedures were identified using the Swedish Classification of Health Care Interventions (KVÅ), with codes beginning with JF (colon and small intestine) or JG (rectum) used to define the surgical cohort (Appendix 1). Surgical site (colonic/small intestinal versus rectal) was included as a covariate. The date of surgery was considered the index date. Patients were excluded if they had undergone any other surgical procedure within one year before or after the index operation to ensure an isolated surgical episode and avoid overlapping postoperative exposure periods, had filled any opioid prescription between 365 and 30 days before surgery in order to ensure opioid naïveté, or died within 365 days after the index operation (Figure 1). All data were pseudonymised prior to analysis.

Covariates were selected a priori based on previous literature and clinical relevance. These included age, sex, American Society of Anaesthesiologists Physical Status Classification (ASA), indication for surgery, surgical site (colonic/small intestinal versus rectal procedures) and duration of surgery (Appendix 2). Body mass index, smoking status, intraoperative blood loss and length of hospital stay were considered as candidate covariates but excluded from the primary model due to high proportions of missing data (20.8%, 69.4%, 37.7% and 83.7%, respectively). As diagnostic data were not consistently available, psychiatric disorders were operationalised using a proxy approach, defined as at least one dispensed prescription within 365 days preceding the index date for medications belonging to the relevant drug category according to the Anatomical Therapeutic Chemical (ATC) classification system. This approach is consistent with prior register‐based studies [18, 19, 20]. Details of the included drugs can be found in appendices (Appendix 3). The primary outcome was NPOU, defined as at least one dispensed prescription for an opioid analgesic (Appendix 3) between 0 and 90 days after the index surgery and at least one dispensed prescription for an opioid analgesic between 90 and 180 days after the index surgery. This definition was chosen to reflect a sustained pattern of postoperative opioid use extending beyond the expected recovery period, as reported previously [2, 3, 4, 5, 6].

Early postoperative opioid dispensing (referred to as opioid at discharge) was defined as at least one dispensed opioid prescription (ATC code N02A) within 7 days of the index operation date, as recorded in the National Prescribed Drug Register. Because individual discharge dates could not be reliably determined, a 7‐day post‐index window was used as a pragmatic proxy for the immediate perioperative period.

Each discharge dispensation (0–7 days) was classified by agent, formulation and morphine milligram equivalents (MME) using the factors of Adams et al., with ketobemidone assigned a factor of 1.0 per Nordic convention [21, 22]. At the patient level, exposures were summarised by agent strength, formulation, MME tertile and estimated duration.

Descriptive statistics were used to summarise baseline characteristics. Continuous variables are presented as medians (interquartile ranges) and categorical variables as counts and percentages. Associations between patient and procedure characteristics and the risk of NPOU were examined using multivariable logistic regression, yielding adjusted odds ratios (aORs) with 95% confidence intervals. Hospital was not included as a covariate in the multivariable model, as the large number of individual units precluded stable estimation within a standard logistic regression framework; hospital‐level variation was instead examined in separate univariable analyses. Statistical significance was defined as a two‐sided p‐value <0.05. As this was a population‐based register study, no a priori sample size calculation was performed; all eligible patients during the study period were included. All analyses were performed using R version 4.3.2 (R Foundation for Statistical Computing, Vienna, Austria).

Data were analysed between May 1st 2025 and November 30th 2025. The study was reported in accordance with the Strengthening the Reporting of Observational Studies in Epidemiology (STROBE) guidelines [23]. The study was approved by the Swedish Ethical Review Authority (reference number 2023–00275‐01), which waived the requirement for informed consent as research was based on pseudonymised register data.

## RESULTS

The analytic cohort comprised 14,233 opioid‐naïve patients undergoing elective colorectal surgery (Table 1, Figure 1). Of these, 374 (2.63%; 95% CI, 2.37%–2.90%) developed NPOU. Several factors were independently associated with the development of NPOU (Table 2). Duration of surgery (aOR, 1.19 per hour; 95% CI, 1.13–1.26), preoperative exposure to non‐opioid analgesics (aOR, 1.90; 95% CI, 1.52–2.37) and sedatives or anxiolytics (aOR, 1.66; 95% CI, 1.31–2.09) were associated with higher odds of NPOU, whereas male sex was associated with lower odds (aOR, 0.76; 95% CI, 0.61–0.95). Receipt of an opioid prescription within 7 days of surgery was also independently associated with persistent use (aOR, 2.19; 95% CI, 1.76–2.72). ASA class was associated with NPOU. Compared with ASA 1, ASA 3 (aOR, 1.89; 95% CI, 1.26–2.90) and ASA 4 (aOR, 3.29; 95% CI, 1.63–6.36) were both associated with increased odds of NPOU, whereas ASA 2 did not differ significantly from ASA 1.

**TABLE 1:** Cohort characteristics overall and by new persistent opioid use.

			New persistent opioid use		*p* ^b^
Group	Characteristic	Overall^a^	No *N* = 13,859^a^	Yes *N* = 374^a^	
Demographics	Age (years)	72.0 (62.0, 79.0)	72.0 (62.0, 79.0)	73.0 (62.0, 79.0)	0.4
	Sex				0.15
	F	7,146 (50%)	6,940 (50%)	206 (55%)	
	M	7,052 (50%)	6,885 (50%)	167 (45%)	
	Unknown	35 (0.2%)	34 (0.2%)	1 (0.3%)	
	Body Mass Index	25.7 (23.1, 28.9)	25.6 (23.0, 28.8)	26.4 (23.7, 29.5)	0.008
	Unknown	2,958	2,881	77	
Clinical characteristics	ASA class				<0.001
	ASA 1	1,665 (12%)	1,632 (12%)	33 (8.8%)	
	ASA 2	8,082 (57%)	7,912 (57%)	170 (45%)	
	ASA 3	4,095 (29%)	3,941 (28%)	154 (41%)	
	ASA 4	239 (1.7%)	225 (1.6%)	14 (3.7%)	
	Unknown	152 (1.1%)	149 (1.1%)	3 (0.8%)	
	Indication				0.2
	Cancer	8,205 (58%)	7,973 (58%)	232 (62%)	
	IBD	715 (5.0%)	693 (5.0%)	22 (5.9%)	
	Diverticulosis	442 (3.1%)	433 (3.1%)	9 (2.4%)	
	Other	4,871 (34%)	4,760 (34%)	111 (30%)	
	Nonopioid analgesics, 1 year preoperatively	3,376 (24%)	3,225 (23%)	151 (40%)	<0.001
	Sedatives or anxiolytics, 1 year preoperatively	3,179 (22%)	3,050 (22%)	129 (34%)	<0.001
	Antidepressants/psychoanaleptics, 1 year preoperatively	1,994 (14%)	1,926 (14%)	68 (18%)	0.016
Perioperative variables	Operation time (min)	177.0 (127.0, 251.0)	177.0 (127.0, 250.0)	201.0 (141.0, 308.0)	<0.001
	Surgical approach				0.3
	Minimally invasive	7,432 (52%)	7,253 (52%)	179 (48%)	
	Open	5,925 (42%)	5,759 (42%)	166 (44%)	
	Converted to open	550 (3.9%)	531 (3.8%)	19 (5.1%)	
	Other	326 (2.3%)	316 (2.3%)	10 (2.7%)	
	Procedure group				0.5
	Colon and small bowel	10,825 (76%)	10,546 (76%)	279 (75%)	
	Rectum	3,408 (24%)	3,313 (24%)	95 (25%)	
	Other	0 (0%)	0 (0%)	0 (0%)	
	Opioid rx filled at discharge				<0.001
	No opioid at discharge	8,441 (59%)	8,276 (60%)	165 (44%)	
	Opioid at discharge	5,792 (41%)	5,583 (40%)	209 (56%)	

^a^ Median (Q1, Q3); *n* (%).

^b^ Wilcoxon rank‐sum test for continuous variables; chi‐squared test for categorical variables.

**TABLE 2:** Multivariable logistic regression — predictors of new persistent opioid use.

Group	Predictor	OR	95% CI	*p*
Demographics	Age (per year)	1.00	0.99, 1.01	0.8
	Sex (ref: Female)			
	Female	—	—	
	Male	0.76	0.61, 0.95	0.016
Clinical characteristics	ASA class (ref: ASA 1)			
	ASA 1	—	—	
	ASA 2	1.00	0.69, 1.50	>0.9
	ASA 3	1.89	1.26, 2.90	0.003
	ASA 4	3.29	1.63, 6.36	<0.001
	Indication (ref: Other)			
	Other	—	—	
	IBD (UC/Crohn)	1.22	0.72, 1.99	0.4
	Diverticulosis	0.75	0.35, 1.43	0.4
	Cancer	1.08	0.86, 1.38	0.5
	Nonopioid analgesics, 1 year preoperatively			
	No	—	—	
	Yes	1.90	1.52, 2.37	<0.001
	Sedatives or anxiolytics, 1 year preoperatively			
	No	—	—	
	Yes	1.66	1.31, 2.09	<0.001
	Antidepressants/psychoanaleptics, 1 year preoperatively			
	No	—	—	
	Yes	0.99	0.74, 1.32	>0.9
Perioperative variables	Operation time (per hour)	1.19	1.13, 1.26	<0.001
	Procedure group			
	Colon/small bowel (JF)	—	—	
	Rectum (JG)	0.94	0.72, 1.22	0.6
	Opioid rx filled at discharge			
	No	—	—	
	Yes	2.19	1.76, 2.72	<0.001

Abbreviations: CI, Confidence Interval, OR, Odds Ratio.

The multivariable model included 14,046 patients (187 excluded due to missing data for sex [*n* = 35] and ASA classification [*n* = 152]). The model's ability to distinguish patients who developed NPOU from those who did not was moderate, with an area under the receiver operating characteristic curve (AUC) of 0.71, where 0.50 represents chance, and 1.0 represents perfect discrimination.

Among discharge‐opioid recipients, unadjusted NPOU rates ranged from 3.4% to 4.4% across strata of formulation, dispensed quantity and estimated duration, with overlapping confidence intervals and no consistent dose–response (Appendix 5). Almost all recipients received a strong opioid (only 7 received a weak opioid), so an agent‐strength contrast was not estimable. The only material difference was binary: recipients versus non‐recipients (3.6% vs. 2.0%). Diagnosis, age, use of antidepressant drugs and site of surgery (colon/small bowel vs. rectal procedures) were not independently associated with NPOU.

In an exploratory univariable analysis, the incidence of NPOU varied across Swedish hospitals with at least four NPOU events, ranging from 1.20% (*n* = 502; OR 0.44 [95% CI, 0.17–0.90] vs. all other hospitals) to 5.97% (*n* = 67; OR 2.37 [95% CI, 0.72–5.77] vs. all other hospitals, Appendix 4). These estimates were unadjusted, and per‐centre event counts were small, yielding wide confidence intervals. We also observed a marked variability across individual surgical interventions. Considering only those procedures, the incidence ranged from 8.9% for ‘colectomy with ileostomy’ (JFH10; *n* = 101 OR 3.62 [95% CI, 1.72–6.98] vs. all other procedures) to 0% for ‘other colostomy’ procedures (JFF30; no NPOU events occurred (0/42) vs. all other procedures) (Appendix 4).

## DISCUSSION AND CONCLUSIONS

In this nationwide cohort of opioid‐naïve patients undergoing elective colorectal surgery in Sweden, 2.6% developed NPOU. This is consistent with the 3.1% reported by Clarke et al. among opioid‐naïve patients aged ≥66 years undergoing major elective surgery, the closest population‐level comparator, which applied the same two‐window definition, and at the lower end of the 3.9–14.0% range reported across 22 mostly US surgical cohorts in a recent systematic review [2, 4, 24, 25]. The frequently cited ~6% of Brummett et al. is not directly comparable, as it was measured only among opioid‐naïve patients who filled a perioperative opioid prescription rather than across all opioid‐naïve patients; the corresponding recipient rate in the present cohort was 3.6% [5]. The discrepancy may reflect a comparatively restrictive opioid stewardship and widespread implementation of ERAS protocols in Sweden, rather than a true difference in patient risk profiles. Cohort selection may also contribute: restricting to opioid‐naïve patients without concurrent procedures isolates the contribution of a single colorectal episode, a design criterion not consistently applied previously.

The most comparable study is the nationwide Austrian cohort by Bologheanu et al., which reported an NPOU incidence of 2.2% following colectomy [3]. That study, however, included patients with prior opioid use and those undergoing concurrent surgical procedures, whereas the present cohort applied strict opioid‐naïvety criteria and excluded overlapping surgical episodes, suggesting the present estimate more closely reflects surgery‐attributable NPOU. Using the same 91–180‐day outcome window, Gong et al. reported persistent opioid use in 4.3% of opioid‐naïve New Zealand patients dispensed an opioid within 7 days of discharge; the comparable figure in the present cohort, NPOU among discharge‐opioid recipients, was 3.6% [7]. Gong's higher headline incidence (9.1%) reflects a longer window (91–365 days) restricted to discharge‐opioid recipients rather than all opioid‐naïve patients. The factor most strongly associated with NPOU in the present study was early postoperative opioid dispensing, with patients receiving an opioid prescription within 7 days of surgery having more than twice the adjusted odds of developing NPOU (aOR 2.19; 95% CI 1.76–2.72), consistent with prior analyses showing that opioids dispensed at discharge independently predict persistent use [26, 27]. The present study extends these observations to a colorectal surgery cohort using national dispensing data and identifies the binary discharge prescription decision rather than cumulative dose, as the clinically relevant unit of analysis. If the discharge prescription is by itself a driver of persistence, opioid‐sparing discharge strategies might become a modifiable intervention point: McKie et al. demonstrated that it is possible to reduce long‐term opioid use from 16% to 11% at 6 months in opioid‐naïve colorectal surgery patients by progressive ERAS implementation [9]. Multimodal opioid‐sparing represents a well‐established alternative; a previous study from our group demonstrated that a structured perioperative bundle significantly reduced in‐hospital opioid use without compromising pain control at a Swedish colorectal unit [28].

Duration of surgery was also independently associated with NPOU, with each additional hour of operating time increasing the odds by 19%. Greater surgical magnitude is associated with amplified inflammatory responses and higher acute postoperative opioid requirements [29, 30, 31], which may sensitise the pain pathways underlying NPOU. From a surgical perspective, minimising operating time where feasible and optimising perioperative analgesia for longer procedures may represent actionable targets.

Preoperative use of non‐opioid analgesics and sedatives or anxiolytics was both independently associated with NPOU, whereas antidepressant use was not. The association with non‐opioid analgesics (aOR 1.90; 95% CI 1.52–2.37) likely reflects underlying chronic pain conditions that are incompletely captured by diagnostic codes in register data. Similarly, preoperative sedative or anxiolytic use (aOR 1.66; 95% CI 1.31–2.09) points to anxiety and sleep disturbance as vulnerability factors, which is consistent with the broader literature on psychological predictors of chronic pain. The absence of an association between antidepressant use and NPOU is consistent with Robertson et al., who found antidepressants to be associated with short‐term opioid refill but not persistent opioid use after lumbar surgery [11]. In the current study, higher ASA classification showed a graded association with NPOU risk, with ASA 4 patients having more than three times the odds compared with ASA 1 (aOR 3.29; 95% CI 1.63–6.36).

IBD and diverticular disease are associated with elevated opioid use during active disease, but surgical diagnosis was not independently associated with NPOU in the present cohort after adjustment [32]. Surgery for malignancy has been associated with a particularly high risk of new persistent opioid use; Khalil et al. found that advanced malignant disease stage increased the adjusted odds of developing NPOU more than fourfold [8]. Cancer constituted 58% of the current cohort, yet carried no excess risk once operating time, early opioid dispensing and ASA class were accounted for.

Substantial variation in NPOU incidence was observed across Swedish hospitals, ranging from 1.2% to 6.0% among hospitals with at least four NPOU events. This unadjusted variation suggests that local practice patterns, including prescribing culture and follow‐up routines, may contribute to NPOU risk beyond patient‐level factors. Because these analyses were unadjusted and hospital was not included in the multivariable model, this variation may equally reflect differences in case mix, disease complexity and referral patterns between centres rather than prescribing culture alone, and the estimates should be regarded as hypothesis‐generating. Within recipients, no prescription feature (agent strength, formulation, quantity or duration) stratified risk (Appendix 5). Discharge prescribing therefore appears to act as a marker rather than a graded exposure, making the decision to prescribe at all, rather than how much, the more promising intervention target.

The principal strengths of this study are its nationwide scope and reliance on prospectively collected, individually linked national registers, which minimise selection bias and provide near‐complete ascertainment of dispensed opioids without dependence on recall. The opioid‐naïve cohort was defined stringently, and new persistent use was identified using a two‐window definition spanning the early and late postoperative periods, reducing misclassification of transient use as persistent. Linkage further enabled adjustment for operating time, ASA class, discharge opioid dispensing and preoperative psychotropic medication.

### This study has several limitations that should be acknowledged

First, its observational design precludes causal inference, and despite multivariable adjustment, residual confounding cannot be excluded.

Second, psychiatric comorbidity was operationalised using dispensed prescriptions for antidepressants, sedatives and anxiolytics as proxy measures. Although this approach is commonly used in register‐based studies, it may incompletely capture underlying mental health conditions, including untreated or non‐pharmacologically treated psychiatric disorders, resulting in potential residual confounding.

Third, both opioid exposure and the NPOU outcome were ascertained from dispensed prescription data, which confirms that a medication was collected but not that it was consumed; NPOU should therefore be interpreted as a dispensing‐based surrogate for sustained use. Two features mitigate this: the two‐window definition means a single over‐supplied prescription cannot alone produce a positive classification, and dispensing data are recorded prospectively, free of recall bias. We could not, however, capture inpatient administration or verify adherence, and because postoperative doses typically exceed one WHO‐defined daily dose (DDD), DDD‐based estimates of duration are overstated—a limitation shared by all comparator studies using dispensing or claims data.

Fourth, discharge opioid prescription was proxied by a dispensation within 7 days of surgery, as discharge dates were not consistently recorded; a 3‐day window yielded a non‐significant association (OR 1.15; 95% CI 0.83–1.57), too narrow to capture the typical timing of the discharge prescription.

Fifth, tumour stage was not available for adjustment. Disease stage influences adjuvant treatment and recovery, and its omission is a potential source of residual confounding, given that cancer was the indication in 58% of the cohort.

Sixth, model discrimination was moderate (AUC, 0.71); accordingly, the model is intended to identify factors associated with NPOU rather than to serve as a validated clinical prediction tool.

Finally, the findings reflect colorectal surgical practice in Sweden, a healthcare system characterised by relatively restrictive opioid prescribing and widespread ERAS implementation. As such, the absolute incidence of NPOU and the relative importance of identified risk factors may differ in healthcare systems with other prescribing routines or organisational structures.

In this nationwide cohort from a setting of restrictive opioid prescribing, approximately one in thirty‐eight opioid‐naïve patients developed NPOU after colorectal surgery. Longer operating time, preoperative use of non‐opioid analgesics or sedatives/anxiolytics, higher ASA score and receipt of an opioid prescription within 7 days of surgery were independently associated with NPOU. Identifying at‐risk patients from routine perioperative characteristics may enable targeted opioid‐sparing measures and structured follow‐up, while national register data can quantify regional variation and benchmark such interventions, informing wider efforts toward safer postoperative prescribing.

## AUTHOR CONTRIBUTIONS

**Claes Gedda:** Conceptualization; investigation; funding acquisition; writing – original draft; methodology; validation; project administration. **Erik Sturegård:** Conceptualization; writing – review and editing. **Mattias Soop:** Conceptualization; investigation; funding acquisition; writing – review and editing; project administration. **Jonas Nygren:** Conceptualization; writing – review and editing. **Anders Thorell:** Conceptualization; writing – review and editing. **Yunzhang Wang:** Formal analysis; software; data curation.

## CONFLICT OF INTEREST STATEMENT

The authors declare no conflicts of interest.

## ETHICS STATEMENT

The study was conducted in accordance with the Declaration of Helsinki and approved by the Swedish Ethical Review Authority (Dnr 2023‐00275‐01). The requirement for informed consent was waived as the study was based on pseudonymised national register data.

## Supporting information


**Appendix 1.** Classification of Health Care Interventions JF & JG (KVÅ).

**Appendix 2.** Definition and sourcing of variables.

**Appendix 3.** Opioids included in database.

**Appendix 4.** Univariable analysis.

**Appendix 5.** New persistent opioid use (NPOU) among discharge‐opioid recipients stratified by agent strength, formulation, dispensed quantity and duration of supply.

**Data S1.** STROBE Checklist—Cohort Study.

## Data Availability

The data that support the findings of this study are available on request from the corresponding author. The data are not publicly available due to privacy or ethical restrictions.
