# Supplementary material for: New persistent opioid use after colorectal surgery in Sweden: A nationwide register‐based cohort study
Source: Colorectal Dis. 2026 Aug 24;28(9):e70601. doi: 10.1111/codi.70601 (PMC13504069; doi:10.1111/codi.70601)
Supplement: Supplementary file 1 [file CODI-28-0-s001.docx]

Appendix 1

Classification of Health Care Interventions JF & JG (KVÅ)

JFA00 Enterotomy

JFA02 Endoscopic removal of foreign body in small intestine

JFA05 Endoscopic polypectomy in small intestine

JFA10 Colotomy

JFA12 Endoscopic removal of foreign body in colon

JFA15 Endoscopic polypectomy in colon

JFA16 Wall biopsy of colon without colotomy

JFA17 Laparoscopic wall biopsy of colon without colotomy

JFA22 Endoscopic injection therapy in small intestine

JFA25 Endoscopic coagulation in small intestine

JFA28 Endoscopic laser therapy in small intestine

JFA32 Other endoscopic hemostatic procedure in small intestine

JFA35 Other endoscopic local procedure with diathermy or heat in small intestine

JFA38 Endoscopic dilation of small intestine

JFA42 Endoscopic injection therapy in colon

JFA45 Endoscopic coagulation in colon

JFA48 Endoscopic laser therapy in colon

JFA52 Other endoscopic hemostatic procedure in colon

JFA55 Other endoscopic local procedure with diathermy or heat in colon

JFA58 Endoscopic dilation of colon

JFA60 Strictureplasty in small intestine

JFA63 Strictureplasty in colon

JFA65 Endoscopic stent placement in small intestine

JFA68 Endoscopic stent placement in colon

JFA70 Enterorrhaphy

JFA71 Laparoscopic enterorrhaphy

JFA73 Excision of local lesion in small intestine

JFA74 Laparoscopic excision of local lesion in small intestine

JFA76 Closure of small bowel fistula

JFA80 Colorrhaphy

JFA81 Laparoscopic colorrhaphy

JFA82 Endoscopic submucosal dissection in colon

JFA83 Excision of local lesion in colon

JFA84 Laparoscopic excision of local lesion in colon

JFA85 Endoscopic mucosal or submucosal resection in colon

JFA86 Closure of colonic fistula

JFA88 Endoscopic submucosal dissection in small intestine

JFA96 Other local intestinal operation

JFA97 Other laparoscopic local operation in small intestine or colon

JFA98 Other transluminal endoscopic local operation in small intestine or colon

JFB00 Small bowel resection

JFB01 Laparoscopic small bowel resection

JFB10 Reversal of small bowel segment

JFB13 Lengthening plasty on small intestine

JFB20 Ileocecal resection

JFB21 Laparoscopic ileocecal resection

JFB30 Right hemicolectomy

JFB31 Laparoscopic right hemicolectomy

JFB33 Other resection involving small intestine and colon

JFB34 Other laparoscopic resection involving small intestine and colon

JFB40 Transverse colon resection

JFB41 Laparoscopic transverse colon resection

JFB43 Left hemicolectomy

JFB44 Laparoscopic left hemicolectomy

JFB46 Sigmoid colon resection

JFB47 Laparoscopic sigmoid colon resection

JFB50 Other colon resection

JFB51 Other laparoscopic colon resection

JFB53 Sigmoid colon resection with simultaneous partial rectal resection

JFB54 Laparoscopic sigmoid resection with simultaneous partial rectal resection

JFB60 Sigmoid colon resection with sigmoidostomy

JFB61 Laparoscopic sigmoid resection with sigmoidostomy and closure of rectal stump

JFB63 Other colon resection with colostomy and closure of distal stump

JFB64 Other laparoscopic colon resection with colostomy and closure of distal stump

JFB96 Other resection of small intestine or colon

JFB97 Other laparoscopic resection of small intestine or colon

JFC01 Laparoscopic enteroanastomosis

JFC10 Ileotransversostomy

JFC11 Laparoscopic ileotransversostomy

JFC20 Other enterocolostomy

JFC21 Other laparoscopic enterocolostomy

JFC30 Colocolostomy

JFC31 Laparoscopic colocolostomy

JFC40 Ileorectal anastomosis

JFC41 Laparoscopic ileorectal anastomosis

JFC50 Colorectal anastomosis

JFC51 Laparoscopic colorectal anastomosis

JFC60 Revision of enteroanastomosis

JFC61 Laparoscopic revision of enteroanastomosis

JFD00 Jejunoileal shunt

JFD03 Duodenal bypass with biliopancreatic diversion

JFD04 Laparoscopic duodenal shunt with biliopancreatic diversion

JFD10 Revision of jejunoileal shunt

JFD13 Revision of duodenal shunt

JFD20 Reversal after jejunoileal shunt

JFD23 Reversal after duodenal shunt

JFD96 Other intestinal shunt operation

JFE00 Small bowel transplantation

JFE96 Other operation related to small bowel transplantation

JFF00 Catheter enterostomy

JFF01 Laparoscopic catheter enterostomy

JFF10 Loop ileostomy

JFF11 Laparoscopic loop ileostomy

JFF13 Enterostomy

JFF20 Cecostomy

JFF21 Laparoscopic cecostomy

JFF23 Transverse colostomy

JFF24 Laparoscopic transverse colostomy

JFF26 Sigmoid colostomy

JFF27 Laparoscopic sigmoid colostomy

JFF30 Other colostomy

JFF31 Other laparoscopic colostomy

JFF40 Appendicostomy

JFF41 Laparoscopic appendicostomy

JFF50 Exteriorization of colonic loop without primary opening

JFF51 Laparoscopic exteriorization of colonic loop without primary opening

JFF60 Opening of exteriorized colonic loop

JFF96 Other exteriorization of small intestine or colon

JFF97 Other laparoscopic exteriorization of small intestine or colon

JFG00 Closure of loop enterostomy without bowel resection

JFG10 Closure of loop colostomy without bowel resection

JFG20 Closure of enterostomy with bowel resection

JFG23 Closure of enterostomy with anastomosis to small intestine

JFG26 Closure of enterostomy with anastomosis to colon

JFG29 Closure of enterostomy with anastomosis to rectum

JFG30 Closure of colostomy with bowel resection

JFG33 Closure of colostomy with anastomosis to colon

JFG36 Closure of colostomy with anastomosis to rectum

JFG40 Revision of entero- or colostomy without laparotomy

JFG50 Laparotomy with revision of entero- or colostomy

JFG53 Revision of ileal pouch

JFG56 Revision of colonic pouch

JFG60 Conversion of conventional ileostomy to continent ileostomy

JFG70 Conversion of continent ileostomy to conventional ileostomy

JFG73 Excision of ileal pouch

JFG76 Excision of colonic pouch with colorectal or coloanal anastomosis

JFG80 Excision of ileal pouch with construction of new continent ileostomy

JFG83 Excision of colonic pouch and construction of new pouch

JFG86 Excision of ileal pouch and construction of new pouch

JFG96 Other operation on stoma or pouch

JFH00 Colectomy with ileorectal anastomosis

JFH01 Laparoscopic colectomy with ileorectal anastomosis

JFH10 Colectomy and ileostomy

JFH11 Laparoscopic colectomy and ileostomy

JFH20 Proctocolectomy with ileostomy

JFH30 Colectomy rectal mucosectomy and ileoanal anastomosis without ileostomy

JFH31 Laparoscopic colectomy rectal excision and ileoanal anastomosis without ileostomy

JFH33 Colectomy rectal mucosectomy ileoanal anastomosis and ileostomy

JFH40 Proctocolectomy with continent ileostomy

JFH96 Other colectomy

JFJ00 Cecopexy

JFJ01 Laparoscopic cecopexy

JFJ96 Other fixation of intestine to abdominal wall

JFJ97 Other laparoscopic fixation of intestine to abdominal wall

JFK00 Division of adhesion

JFK01 Laparoscopic division of adhesion

JFK10 Adhesiolysis

JFK20 Adhesiolysis with small intestinal fixation

JFK96 Other division of adhesions for bowel obstruction

JFK97 Other laparoscopic division of adhesions for bowel obstruction

JFL00 Laparotomy and desinvagination of intestine

JFL10 Laparotomy and reposition or detorsion of intestine

JFL11 Laparoscopic reposition or manipulation of obstructed intestine

JFL20 Manipulation of obstructing intestinal content

JFL96 Other treatment of intestinal obstruction without resection or adhesiolysis

JFM00 Intraoperative bowel lavage

JFW96 Other operation on small intestine or colon

JFW97 Other laparoscopic operation on small intestine or colon

JFW98 Other transluminal endoscopic operation on small intestine or colon

JGA00 Proctotomy

JGA02 Endoscopic removal of foreign body in rectum

JGA05 Endoscopic polypectomy in rectum

JGA22 Endoscopic injection therapy in rectum

JGA28 Endoscopic coagulation in rectum

JGA32 Endoscopic laser therapy in rectum

JGA35 Other endoscopic hemostatic procedure in rectum

JGA48 Endoscopic submucosal dissection in rectum

JGA52 Other endoscopic local procedure with diathermy or heat in rectum

JGA58 Endoscopic dilation of rectum

JGA60 Suture of rectum

JGA68 Endoscopic stent placement in rectum

JGA70 Proctotomy with excision of lesion in rectum

JGA73 Transanal excision of lesion in rectum

JGA75 Endoscopic microsurgical excision of lesion in rectum

JGA76 Stapled transanal rectal resection

JGA96 Other proctotomy or local rectal operation

JGA97 Other laparoscopic proctotomy or local rectal operation

JGA98 Other transluminal endoscopic local rectal operation

JGB00 Rectal resection with total mesorectal excision

JGB01 Laparoscopic rectal resection with total mesorectal excision

JGB03 Rectal resection with partial mesorectal excision

JGB04 Laparoscopic rectal resection with partial mesorectal excision

JGB10 Rectal resection with sigmoidostomy and closure of rectal stump

JGB11 Laparoscopic rectal resection with sigmoidostomy and closure of rectal stump

JGB20 Rectal resection with abdominoperineal pull-through anastomosis

JGB30 Abdominoperineal rectal amputation

JGB31 Laparoscopic abdominoperineal rectal amputation

JGB33 Abdominoperineal rectal amputation with intersphincteric dissection

JGB34 Laparoscopy-assisted abdominoperineal rectal amputation with intersphincteric dissection

JGB36 Extended rectal excision

JGB40 Rectal extirpation and ileostomy

JGB50 Mucosectomy and ileoanal anastomosis

JGB60 Extirpation of rectum or ileoanal anastomosis

JGB61 Laparoscopic excision of rectum and ileoanal anastomosis after previous colectomy

JGB96 Other rectal resection or extirpation

JGB97 Other laparoscopic rectal resection or extirpation

JGC00 Rectopexy

JGC01 Laparoscopic rectopexy

JGC10 Perineal rectopexy

JGC20 Transanal suture

JGC30 Excision and suture of rectal mucosa

JGC40 Anorectal reconstruction for anal atresia

JGC96 Other rectal reconstruction

JGC97 Other laparoscopic rectal reconstruction

JGD00 Excision of perineal tumor recurrence

JGW96 Other operation on rectum

JGW97 Other laparoscopic operation on rectum

JGW98 Other transluminal endoscopic operation on rectum
