# Supplementary material for: New persistent opioid use after colorectal surgery in Sweden: A nationwide register‐based cohort study
Source: Colorectal Dis. 2026 Aug 24;28(9):e70601. doi: 10.1111/codi.70601 (PMC13504069; doi:10.1111/codi.70601)
Supplement: Supplementary file 2 [file CODI-28-0-s002.docx]

**Appendix 5.** New persistent opioid use (NPOU) among discharge-opioid recipients stratified by agent strength, formulation, dispensed quantity, and duration of supply.

| **Stratification** | **Stratum** | **n** | **NPOU events** | **NPOU, % (95% CI)** |
| --- | --- | --- | --- | --- |
| **Overall** | | | | |
|  | All opioid-naïve patients | 14,233 | 374 | 2.6 (2.4–2.9) |
| **Discharge opioid,** *any N02A dispensing within 7 days of index surgery* | | | | |
|  | Recipient | 5,792 | 209 | 3.6 (3.2–4.1) |
|  | Non-recipient | 8,441 | 165 | 2.0 (1.7–2.3) |
| **Agent strength** | | | | |
|  | Strong opioid | 5,776 | 208 | 3.6 (3.2–4.1) |
|  | Weak opioid | 7 | 0 | 0.0 (0.0–35.4) |
|  | *Unclassifiedᵇ* | *9* | *1* | *11.1 (2.0–43.5)* |
|  | Non-recipient | 8,441 | 165 | 2.0 (1.7–2.3) |
| **Formulation** | | | | |
|  | Any extended-release | 4,566 | 154 | 3.4 (2.9–3.9) |
|  | Immediate-release only | 1,217 | 54 | 4.4 (3.4–5.7) |
|  | *Unclassifiedᵇ* | *9* | *1* | *11.1 (2.0–43.5)* |
|  | Non-recipient | 8,441 | 165 | 2.0 (1.7–2.3) |
| **Dispensed quantity,** *morphine milligram equivalents (MME)ᵃ* | | | | |
|  | Tertile 1: ≤209 mg MME | 2,074 | 71 | 3.4 (2.7–4.3) |
|  | Tertile 2: 210–315 mg MME | 2,206 | 79 | 3.6 (2.9–4.4) |
|  | Tertile 3: >315 mg MME | 1,503 | 58 | 3.9 (3.0–5.0) |
|  | *Unclassifiedᵇ* | *9* | *1* | *11.1 (2.0–43.5)* |
|  | Non-recipient | 8,441 | 165 | 2.0 (1.7–2.3) |
| **Duration of supply,** *defined daily doses (DDD-days)ᶜ* | | | | |
|  | Short: ≤3 DDD-days | 4,298 | 149 | 3.5 (3.0–4.1) |
|  | Medium: 4–7 DDD-days | 1,305 | 51 | 3.9 (3.0–5.1) |
|  | Long: >7 DDD-days | 180 | 8 | 4.4 (2.3–8.5) |
|  | *Unclassifiedᵇ* | *9* | *1* | *11.1 (2.0–43.5)* |
|  | Non-recipient | 8,441 | 165 | 2.0 (1.7–2.3) |

*NPOU, new persistent opioid use (at least one opioid dispensation 0–90 days post-surgery and at least one dispensation 91–180 days post-surgery). CI, confidence interval. MME, morphine milligram equivalents. DDD, defined daily dose. Confidence intervals are Wilson score 95% CIs.*

*ᵃ MME was calculated as DDD-days × drug-specific mg-MME conversion factor per DDD-day: oxycodone 112.5 mg/DDD-day (75 mg DDD × 1.5), morphine 100 mg/DDD-day (0.1 g DDD × 1000 × 1.0), ketobemidone 50 mg/DDD-day, fentanyl patch 120 mg/DDD-day, tramadol 60 mg/DDD-day, and codeine combination products 3.6–13.5 mg/DDD-day depending on codeine content. DDD values for morphine and tramadol are registered in grams in the WHO ATC/DDD index and were converted to mg before applying conversion factors. Tertile boundaries were derived from the recipient distribution (T1 ≤209 mg, T2 210–315 mg, T3 >315 mg).*

*ᵇ Nine recipients (1 with NPOU) could not be classified in the strata above because no MME value could be derived (every dispensation was a part-package record, with a dispensed quantity below one package). They are included in the recipient total (n = 5,792) but excluded from the strength, formulation, quantity, and duration comparisons. The non-recipient reference group (n = 8,441; 165 NPOU) corresponds to the "No opioid at discharge" group in Table 1.*

*ᶜ Duration was estimated as DDD-days supplied per dispensation (forpddd field, Swedish Prescribed Drug Register). Short ≤3 DDD-days; medium 4–7 DDD-days; long >7 DDD-days.*

*P-values for comparisons within discharge-opioid recipients: formulation p = 0.09; dispensed quantity p = 0.79; duration of supply p = 0.62 (chi-squared). Agent strength was not formally compared, as only 7 recipients received a weak opioid. No drug-level characteristic was significantly associated with NPOU risk within the recipient group.*
