# Supplementary material for: New persistent opioid use after colorectal surgery in Sweden: A nationwide register‐based cohort study
Source: Colorectal Dis. 2026 Aug 24;28(9):e70601. doi: 10.1111/codi.70601 (PMC13504069; doi:10.1111/codi.70601)
Supplement: Supplementary file 3 [file CODI-28-0-s003.docx]

Appendix 2

Definition and sourcing of variables

| **Variable** | **Source** | **Definition/unit** |
| --- | --- | --- |
| Date of index-surgery | SPOR | First instance of a surgical procedure registered in SPOR |
| Age | SPOR | Years old at time of index-surgery |
| Sex | SPOR | Sex(male/female) |
| ASA class | SPOR | ASA class 1-5 sourced at date of index-surgery |
| BMI | SPOR | Weight and height sourced at date of index-surgery |
| Surgical indication | SPOR | ICD-10 code in main diagnosis corresponding to cancer (C*), Inflammatory Bowel Disease (K50* & K51*), Diverticulitis(K57) or other respectively |
| Surgical approach | SPOR | Minimal invasive or open. Robotic surgery classified as minimally invasive |
| Duration of surgery | SPOR | Minutes |
| Bleeding volume | SPOR | Milliliters |
| Preoperative use of antidepressant/psychoanaleptic drugs | NPDR | ATC code = N06* 365-0 days before surgery |
| Preoperative use of neuroleptic/sedative/anxiolytic drugs | NPDR | ATC code = N05* 365-0 days before surgery |
| Opioid prescription filled at discharge | NPDR | Prescription issued within +/- 2 days of discharge, subsequently filled |
| Preoperative use of opioids | NPDR | ATC code = N02A* 365-30 days before surgery |
| New Persistent Opioid Use | NPDR | ATC code = N02A* at least once 0-90 days after index surgery AND  ATC code = N02A* at least once 91-180 days after index surgery |
| Substance-use disorders | NPR | Main or concomitant diagnosis 365-0 days before surgery |
| Death | NCDR | Death within 365 days after index-surgery |
|  |  |  |
|  |  |  |
|  |  |  |
|  |  |  |
| **Legend**  SPOR – Swedish Perioperative Register  NPDR – National Public Drug Register  NPR – National Patient Register  NCR – National Cause of Death Register |  |  |
