# Supplementary material for: New persistent opioid use after colorectal surgery in Sweden: A nationwide register‐based cohort study
Source: Colorectal Dis. 2026 Aug 24;28(9):e70601. doi: 10.1111/codi.70601 (PMC13504069; doi:10.1111/codi.70601)
Supplement: Supplementary file 4 [file CODI-28-0-s004.docx]

Opioids included in database

| **ATC Code** | **Drug name** |
| --- | --- |
| N02AA01 | Morphine |
| N02AA03 | Hydromorphone |
| N02AA05 | Oxycodone |
| N02AA55 | Oxycodone (combinations) |
| N02AB01 | Ketobemidone |
| N02AB02 | Pethidine (Meperidine) |
| N02AB03 | Fentanyl |
| N02AD01 | Pentazocine |
| N02AE01 | Buprenorphine |
| N02AG01 | Morphine + antiemetic |
| N02AG02 | Ketobemidone (combinations) |
| N02AG04 | Hydromorphone(cominations) |
| N02AJ06/09 | Codein(combinations) |
| N02AJ13 | Tramadol(combinations) |
| N02AX02 | Tramadol |
| N02AX06 | Tapentadol |

Drugs defined as antidepressant/psychoanaleptic drugs

| **ATC Code** | **Drug name** |
| --- | --- |
| N06AA01 | Desipramine |
| N06AA02 | Imipramine |
| N06AA03 | Imipramine |
| N06AA04 | Clomipramine |
| N06AA05 | Opipramol |
| N06AA06 | Trimipramine |
| N06AA07 | Lofepramine |
| N06AA08 | Dibenzepin |
| N06AA09 | Amitriptyline |
| N06AA10 | Nortriptyline |
| N06AA11 | Protriptyline |
| N06AA12 | Doxepin |
| N06AA13 | Iprindole |
| N06AA14 | Melitracen |
| N06AA15 | Butriptyline |
| N06AA16 | Dosulepin |
| N06AA17 | Amoxapine |
| N06AA18 | Dimetacrine |
| N06AA19 | Amineptine |
| N06AA21 | Maprotiline |
| N06AA23 | Quinupramine |
| N06AB02 | Zimelidine |
| N06AB03 | Fluoxetine |
| N06AB04 | Citalopram |
| N06AB05 | Paroxetine |
| N06AB06 | Sertraline |
| N06AB07 | Alaproclate |
| N06AB08 | Fluvoxamine |
| N06AB09 | Etoperidone |
| N06AB10 | Escitalopram |
| N06AF01 | Isocarboxazid |
| N06AF02 | Nialamide |
| N06AF03 | Phenelzine |
| N06AF04 | Tranylcypromine |
| N06AF05 | Iproniazid |
| N06AF06 | Iproclozide |
| N06AG02 | Moclobemide |
| N06AG03 | Toloxatone |
| N06AX01 | Oxitriptan |
| N06AX02 | Tryptophan |
| N06AX03 | Mianserin |
| N06AX04 | Nomifensine |
| N06AX05 | Trazodone |
| N06AX06 | Nefazodone |
| N06AX07 | Minaprine |
| N06AX08 | Bifemelane |
| N06AX09 | Viloxazine |
| N06AX10 | Oxaflozane |
| N06AX11 | Mirtazapine |
| N06AX13 | Medifoxamine |
| N06AX14 | Tianeptine |
| N06AX15 | Pivagabine |
| N06AX16 | Venlafaxine |
| N06AX17 | Milnacipran |
| N06AX18 | Reboxetine |
| N06AX19 | Gepirone |
| N06AX21 | Duloxetine |
| N06AX23 | Desvenlafaxine |
| N06AX26 | Vortioxetine |
| N06BA01 | Amfetamine |
| N06BA02 | Dexamfetamine |
| N06BA03 | Metamfetamine |
| N06BA04 | Methylphenidate |
| N06BA05 | Pemoline |
| N06BA06 | Fencamfamin |
| N06BA07 | Modafinil |
| N06BA08 | Fenozolone |
| N06BA09 | Atomoxetine |
| N06BA10 | Fenetylline |
| N06BA11 | Dexmethylphenidate |
| N06BA12 | Lisdexamfetamine |
| N06BA13 | Armodafinil |
| N06BA14 | Solriamfetol |
| N06BC01 | Caffeine |
| N06BC02 | Propentofylline |
| N06BX01 | Meclofenoxate |
| N06BX02 | Pyritinol |
| N06BX03 | Piracetam |
| N06BX04 | Deanol |
| N06BX05 | Fipexide |
| N06BX06 | Citicoline |
| N06BX07 | Oxiracetam |
| N06BX08 | Pirisudanol |
| N06BX09 | Linopirdine |
| N06BX10 | Nizofenone |
| N06BX11 | Aniracetam |
| N06BX12 | Acetylcarnitine |
| N06BX13 | Idebenone |
| N06BX14 | Prolintane |
| N06BX15 | Pipradrol |
| N06BX16 | Pramiracetam |
| N06BX17 | Adrafinil |
| N06BX18 | Vinpocetine |
| N06DX01 | Memantine |
| N06DX02 | Ginkgo |
| N06DX03 | Aducanumab |
| N06DX04 | Lecanemab |
| N06DX05 | Donanemab |

**Drugs defined as Neuroleptic drugs**

| ATC Code | Drug name |
| --- | --- |
| N05AA01 | Chlorpromazine |
| N05AA02 | Levomepromazine |
| N05AA03 | Promazine |
| N05AA04 | Acepromazine |
| N05AA05 | Triflupromazine |
| N05AB01 | Dixyrazine |
| N05AB02 | Fluphenazine |
| N05AB03 | Perphenazine |
| N05AB04 | Aceperone |
| N05AB05 | Thiopropazate |
| N05AB06 | Trifluoperazine |
| N05AB07 | Acetophenazine |
| N05AB08 | Thiethylperazine |
| N05AB09 | Flupentixol |
| N05AB10 | Zuclopenthixol |
| N05AB11 | Periciazine |
| N05AB12 | Pipothiazine |
| N05AC01 | Periciazine |
| N05AC02 | Thioridazine |
| N05AC03 | Mesoridazine |
| N05AC04 | Pipotiazine |
| N05AD01 | Haloperidol |
| N05AD02 | Trifluperidol |
| N05AD03 | Melperone |
| N05AD04 | Pipamperone |
| N05AD05 | Droperidol |
| N05AE01 | Oxypertine |
| N05AE02 | Molindone |
| N05AE03 | Sertindole |
| N05AF01 | Fluspirilene |
| N05AF02 | Pimozide |
| N05AG01 | Penfluridol |
| N05AH01 | Loxapine |
| N05AH02 | Clozapine |
| N05AH03 | Olanzapine |
| N05AH04 | Quetiapine |
| N05AH05 | Asenapine |
| N05AH06 | Paliperidone |
| N05AH07 | Lurasidone |
| N05AL01 | Sulpiride |
| N05AL02 | Sultopride |
| N05AL03 | Tiapride |
| N05AL04 | Remoxipride |
| N05AL05 | Amisulpride |
| N05AN01 | Lithium |
| N05AX07 | Prothipendyl |
| N05AX08 | Risperidone |
| N05AX09 | Zotepine |
| N05AX10 | Mosapramine |
| N05AX11 | Ziprasidone |
| N05AX12 | Aripiprazole |
| N05AX13 | Paliperidone |
| N05AX14 | Iloperidone |
| N05AX15 | Cariprazine |
| N05AX16 | Brexpiprazole |
| N05BA01 | Diazepam |
| N05BA02 | Chlordiazepoxide |
| N05BA03 | Medazepam |
| N05BA04 | Oxazepam |
| N05BA05 | Potassium clorazepate |
| N05BA06 | Lorazepam |
| N05BA07 | Adinazolam |
| N05BA08 | Bromazepam |
| N05BA09 | Clobazam |
| N05BA10 | Ketazolam |
| N05BA11 | Prazepam |
| N05BA12 | Alprazolam |
| N05BA13 | Halazepam |
| N05BA14 | Pinazepam |
| N05BA15 | Camazepam |
| N05BA16 | Nordazepam |
| N05BA17 | Fludiazepam |
| N05BA18 | Etizolam |
| N05BA19 | Tofisopam |
| N05BB01 | Hydroxyzine |
| N05BB02 | Captodiame |
| N05BC01 | Meprobamate |
| N05BE01 | Buspirone |
| N05CA01 | Pentobarbital |
| N05CA02 | Secobarbital |
| N05CA03 | Butobarbital |
| N05CA04 | Barbital |
| N05CA05 | Amobarbital |
| N05CA06 | Hexobarbital |
| N05CA07 | Talbutal |
| N05CA08 | Vinylbital |
| N05CA09 | Thiopental |
| N05CA10 | Cyclobarbital |
| N05CD01 | Flurazepam |
| N05CD02 | Nitrazepam |
| N05CD03 | Flunitrazepam |
| N05CD04 | Esterazolam |
| N05CD05 | Triazolam |
| N05CD06 | Lormetazepam |
| N05CD07 | Temazepam |
| N05CD08 | Midazolam |
| N05CD09 | Brotizolam |
| N05CD10 | Quazepam |
| N05CD11 | Loprazolam |
| N05CD12 | Zolpidem |
| N05CD13 | Zopiclone |
| N05CD14 | Zaleplon |
| N05CF01 | Zopiclone |
| N05CF02 | Zolpidem |
| N05CF03 | Zaleplon |
| N05CM01 | Methaqualone |
| N05CM02 | Clomethiazole |
| N05CM03 | Bromisoval |
| N05CM04 | Carbromal |
| N05CM05 | Scopolamine |
| N05CM06 | Propiomazine |
| N05CM07 | Triclofos |
| N05CM08 | Chloralhydrate |
