# Supplementary material for: New persistent opioid use after colorectal surgery in Sweden: A nationwide register‐based cohort study
Source: Colorectal Dis. 2026 Aug 24;28(9):e70601. doi: 10.1111/codi.70601 (PMC13504069; doi:10.1111/codi.70601)
Supplement: Supplementary file 5 [file CODI-28-0-s005.docx]

**STROBE Checklist — Cohort Study**

*Gedda C et al. New Persistent Opioid Use After Colorectal Surgery in Sweden: A Nationwide Registry-Based Cohort Study*

Line numbers refer to the extracted plain-text version of Article_final.docx (pandoc export)

| **Item** | **No.** | **Recommendation** | **Location in manuscript (line numbers)** |
| --- | --- | --- | --- |
| **TITLE AND ABSTRACT** | | | |
| Title and abstract | 1a | Indicate the study design with a commonly used term in the title or the abstract | Line 1–2: Title includes 'Nationwide Registry-Based Cohort Study'.  Line 49–50: Abstract Method states 'nationwide retrospective cohort study'. |
|  | 1b | Provide an informative and balanced summary of what was done and what was found | Lines 45–77: Structured abstract covers design, setting, population, outcomes, key results, and implications. |
| **INTRODUCTION** | | | |
| Background/rationale | 2 | Explain the scientific background and rationale for the investigation being reported | Lines 81–131: Global opioid crisis, NPOU as serious postoperative adverse event, emerging international data, gap in Swedish/Nordic evidence, prior Swedish study (Lindeberg et al., ref 15) and its limitations. |
| Objectives | 3 | State specific objectives, including any prespecified hypotheses | Lines 131–133: 'We therefore aim to describe the prevalence of NPOU 6 months after colorectal surgery in Sweden and to identify any associated perioperative factors. We hypothesized that the incidence of NPOU in Sweden would be less than that reported in US cohorts.' |
| **METHODS** | | | |
| Study design | 4 | Present key elements of study design early in the paper | Line 137: 'This nationwide retrospective cohort study used data from multiple Swedish national health registries.' |
| Setting | 5 | Describe the setting, locations, and relevant dates, including periods of recruitment, exposure, follow-up, and data collection | Lines 137–150: Sweden, nationwide. Study period 1 January 2018 – 31 December 2022. Follow-up 180 days post-index (defined by NPOU outcome). Data analyzed 1 May – 30 November 2025 (line 219). Data sources: SPOR, NPDR, NPR, NCDR. |
| Participants | 6a | Give the eligibility criteria, and the sources and methods of selection of participants. Describe methods of follow-up | Lines 152–165: Adults ≥18 years, elective inpatient colorectal surgery (KVÅ JF/JG), Sweden 2018–2022. Exclusions: opioid Rx 365–30 days preoperatively; other surgery within ±1 year; death within 365 days. Source: SPOR linked to NPDR, NPR, NCDR via personal identity number. Follow-up via NPDR dispensing records to 180 days. |
|  | 6b | For matched studies, give matching criteria and number of exposed and unexposed | Not applicable — not a matched study. |
| Variables | 7 | Clearly define all outcomes, exposures, predictors, potential confounders, and effect modifiers. Give diagnostic criteria, if applicable | Lines 167–201: Primary outcome NPOU defined (lines 184–190). Covariates listed (lines 167–182): age, sex, ASA, indication, surgical site, operative time; psychiatric proxy (ATC-based dispensing, lines 175–182); early postoperative opioid dispensing defined (lines 192–201). Appendices 1–3 detail KVÅ codes and drug lists. |
| Data sources / measurement | 8* | For each variable of interest, give sources of data and details of methods of assessment | Lines 137–150: SPOR (operative data incl. KVÅ codes, operative time, ASA); NPDR (dispensed prescriptions by ATC code); NPR (diagnoses, admissions); NCDR (date of death). Opioid at discharge: NPDR, N02A within 7 days (lines 192–201). |
| Bias | 9 | Describe any efforts to address potential sources of bias | Lines 160–165: Opioid-naïvety ensured by 30–365 day preoperative washout. Surgical isolation ensured by excluding concurrent procedures within ±1 year. Lines 197–201: 7-day discharge window justified; sensitivity analysis with 3-day window reported (lines 403–411). |
| Study size | 10 | Explain how the study size was arrived at | Lines 213–215: 'As this was a population-based registry study, no a priori sample size calculation was performed; all eligible patients during the study period were included.' N=14,233 reported in Results (line 228). |
| Quantitative variables | 11 | Explain how quantitative variables were handled in the analyses | Lines 167–171: Operative time treated as continuous (per hour, Table 2). Age treated as continuous (per year, Table 2). ASA categorised as 1/2/3/4 (ref: ASA 1); categories reported in Table 1 (lines 605–645) and Table 2 (lines 820–840). |
| Statistical methods | 12a | Describe all statistical methods, including those used to control for confounding | Lines 203–215: Descriptive statistics (medians, IQR, n, %). Multivariable logistic regression with a priori covariate selection; aORs with 95% CI. AUC reported for model discrimination (lines 245–249). Hospital-level variation in separate univariable models (lines 208–212). |
|  | 12b | Describe any methods used to examine subgroups and interactions | Lines 208–212: Hospital-level univariable logistic models. KVÅ procedure-level subgroup analysis (univariable), reported in Appendix 4 (lines 256–263). No formal interaction testing performed. |
|  | 12c | Explain how missing data were addressed | Lines 172–175: BMI (20.8%), smoking (69.4%), blood loss (37.7%), hospital stay (83.7%) excluded from primary model due to missing data — stated explicitly. Lines 245–247: 187 patients excluded from multivariable model due to missing sex (n=35) or ASA (n=152); complete-case analysis. |
|  | 12d | If applicable, explain how loss to follow-up was addressed | Registry-based study with near-complete follow-up via mandatory NPDR reporting. Patients dying within 365 days excluded at design stage (line 164). No further loss to follow-up expected in Swedish mandatory registers. |
|  | 12e | Describe any sensitivity analyses | Lines 403–411: Sensitivity analysis using 3-day window for opioid-at-discharge variable reported (OR 1.15, 95% CI 0.83–1.57, non-significant), with explanation that median LOS ~5 days accounts for the null result. |
| **RESULTS** | | | |
| Participants | 13* | (a) Report numbers at each stage; (b) reasons for non-participation; (c) consider flow diagram | Lines 228–229: N=14,233 opioid-naïve patients after exclusions. Figure 1 (flow diagram) shows eligible pool, exclusions with reasons (preop opioid use, concurrent surgery, death). Lines 245–247: 187 further excluded from multivariable model (missing sex/ASA).  See also Figure 1. |
| Descriptive data | 14* | (a) Characteristics of participants; (b) missing data for each variable; (c) follow-up time | Table 1 (lines 566–790): Full cohort characteristics stratified by NPOU. BMI missing n=2,958 reported in table. Follow-up 180 days (defined by NPOU outcome definition, lines 184–190). Surgical approach, procedure group, preoperative drug use, opioid at discharge all reported. |
| Outcome data | 15* | Report numbers of outcome events or summary measures over time | Lines 229–230: 374/14,233 (2.63%; 95% CI 2.37–2.90%) developed NPOU. NPOU by hospital (range 1.20–5.97%, lines 256–258) and by KVÅ code (range 0–8.9%, lines 259–263) in Appendix 4. |
| Main results | 16a | Give unadjusted and adjusted estimates and precision; state which confounders were adjusted for and why | Table 2 (lines 793–): aORs with 95% CI for all model covariates. Covariates selected a priori (lines 167–168). Bivariate p-values in Table 1 provide unadjusted comparisons. Results text (lines 230–263) summarises key findings. |
|  | 16b | Report category boundaries when continuous variables were categorised | ASA categories (1, 2, 3, 4) defined per standard ASA classification, reported in Table 1 and Table 2. Operative time and age treated as continuous — no categorisation. |
|  | 16c | Consider translating relative risk estimates into absolute risk | Lines 280–283: Absolute incidence stated as '1 in 38 opioid-naïve patients'. Abstract conclusion (line 69) and Discussion conclusion (line 420) both express finding in absolute terms alongside relative ORs. |
| Other analyses | 17 | Report other analyses — subgroups, interactions, sensitivity analyses | Lines 256–263: Hospital-level variation (univariable ORs per hospital) and KVÅ procedure-level variation, both in Appendix 4. Lines 403–411: Sensitivity analysis with 3-day discharge window. All pre-specified in Methods. |
| **DISCUSSION** | | | |
| Key results | 18 | Summarise key results with reference to study objectives | Lines 267–283: 2.6% NPOU incidence compared to meta-analysis (13.8%, ref 22) and US cohort (6%, ref 5); contextualised to Swedish prescribing environment and cohort design. Lines 420–434: Conclusion paragraph summarises all key predictors. |
| Limitations | 19 | Discuss limitations, including sources of potential bias or imprecision; direction and magnitude | Lines 386–416 (four numbered limitations): (1) observational design, residual confounding; (2) psychiatric proxy via dispensing — incomplete capture of untreated conditions; (3) dispensed Rx ≠ consumption, no inpatient data, exposure misclassification; (4) 7-day discharge proxy with sensitivity analysis; (5) Swedish-specific generalisability. |
| Interpretation | 20 | Give a cautious overall interpretation considering objectives, limitations, and results from similar studies | Lines 267–434: Discussion contextualises findings against Bologheanu (ref 3), Gong (ref 7), McKie (ref 9), Lindeberg (ref 15). Causal language avoided throughout ('associated with', 'may contribute'). Limitations explicitly stated before conclusions. |
| Generalisability | 21 | Discuss the generalisability (external validity) of the results | Lines 412–416: 'The findings reflect colorectal surgical practice in Sweden, a healthcare system characterized by relatively restrictive opioid prescribing and widespread ERAS implementation. As such, the absolute incidence of NPOU and the relative importance of identified risk factors may differ in healthcare systems with other prescribing routines or organizational structures.' |
| **OTHER INFORMATION** | | | |
| Funding | 22 | Give the source of funding and the role of the funders | Lines 300-302 |

** Give information separately for exposed and unexposed groups where applicable.*
