# Supplementary material for: New persistent opioid use after colorectal surgery in Sweden: A nationwide register‐based cohort study
Source: Colorectal Dis. 2026 Aug 24;28(9):e70601. doi: 10.1111/codi.70601 (PMC13504069; doi:10.1111/codi.70601)
Supplement: Supplementary file 6 [file CODI-28-0-s006.docx]

Appendix 4. Univariable analysis

NPOU incidence per hospital, operations n>=40. (Individual hospital vs. all others)

| Hospital | Operations(n) | NPOU(n) | NPOU(%) | OR (95% CI) | p |
| --- | --- | --- | --- | --- | --- |
| Sahlgrenska sjukhuset | 67 | 4 | 5.97 | 2.37 (0.72–5.77) | 0.096 |
| Helsingborgs lasarett | 347 | 20 | 5.76 | 2.34 (1.43–3.62) | <0.001 |
| Norrlands universitetssjukhus | 239 | 12 | 5.02 | 1.99 (1.05–3.44) | 0.022 |
| Höglandssjukhuset Eksjö | 103 | 5 | 4.85 | 1.90 (0.67–4.24) | 0.163 |
| SkaS Lidköping | 211 | 10 | 4.74 | 1.87 (0.92–3.37) | 0.057 |
| Lasarettet Ljungby | 65 | 3 | 4.62 |  |  |
| Centrallasarettet Växjö | 206 | 9 | 4.37 | 1.71 (0.81–3.17) | 0.120 |
| Östra sjukhuset | 653 | 28 | 4.29 | 1.71 (1.13–2.49) | 0.007 |
| Värnamo sjukhus | 49 | 2 | 4.08 |  |  |
| KS Solna | 648 | 26 | 4.01 | 1.59 (1.03–2.34) | 0.025 |
| Mälarsjukhuset | 216 | 8 | 3.70 | 1.43 (0.64–2.74) | 0.322 |
| Danderyds Sjukhus | 662 | 23 | 3.47 | 1.36 (0.86–2.03) | 0.165 |
| SUS Malmö | 609 | 21 | 3.45 | 1.34 (0.83–2.05) | 0.197 |
| Hallands Sjukhus Varberg | 233 | 8 | 3.43 | 1.32 (0.60–2.53) | 0.440 |
| Sundsvalls sjukhus | 205 | 7 | 3.41 | 1.32 (0.56–2.61) | 0.479 |
| Länssjukhuset Ryhov | 239 | 8 | 3.35 | 1.29 (0.58–2.46) | 0.484 |
| SUS Lund | 63 | 2 | 3.17 |  |  |
| Hallands Sjukhus Halmstad | 244 | 7 | 2.87 | 1.10 (0.46–2.17) | 0.812 |
| Hudiksvalls sjukhus | 105 | 3 | 2.86 |  |  |
| Gävle Sjukhus | 391 | 11 | 2.81 | 1.07 (0.55–1.88) | 0.816 |
| Södertälje Sjukhus AB | 73 | 2 | 2.74 |  |  |
| Centralsjukhuset Kristianstad | 369 | 10 | 2.71 | 1.03 (0.51–1.85) | 0.920 |
| Universitetssjukhuset Örebro | 554 | 15 | 2.71 | 1.03 (0.59–1.68) | 0.905 |
| Alingsås Lasarett | 226 | 6 | 2.65 | 1.01 (0.40–2.09) | 0.979 |
| SÄS Borås | 340 | 9 | 2.65 | 1.01 (0.48–1.85) | 0.982 |
| Nyköpings lasarett | 161 | 4 | 2.48 | 0.94 (0.29–2.24) | 0.909 |
| Västmanlands Sjukhus Västerås | 413 | 10 | 2.42 | 0.92 (0.45–1.64) | 0.790 |
| Södersjukhuset | 564 | 13 | 2.30 | 0.87 (0.47–1.46) | 0.625 |
| Kungälvs sjukhus | 261 | 6 | 2.30 | 0.87 (0.34–1.80) | 0.738 |
| Länssjukhuset i Kalmar | 263 | 6 | 2.28 | 0.86 (0.34–1.78) | 0.723 |
| Visby lasarett | 88 | 2 | 2.27 |  |  |
| Örnsköldsviks sjukhus | 137 | 3 | 2.19 |  |  |
| Centralsjukhuset Karlstad | 412 | 8 | 1.94 | 0.73 (0.33–1.38) | 0.379 |
| Ersta sjukhus | 258 | 5 | 1.94 | 0.73 (0.26–1.60) | 0.486 |
| Norra Älvsborgs Länssjukhus | 518 | 10 | 1.93 | 0.72 (0.36–1.29) | 0.314 |
| Lasarettet i Ystad | 639 | 12 | 1.88 | 0.70 (0.37–1.19) | 0.228 |
| Norrtälje Sjukhus | 59 | 1 | 1.69 |  |  |
| Torsby Sjukhus | 66 | 1 | 1.52 |  |  |
| Sunderby Sjukhus | 355 | 5 | 1.41 | 0.52 (0.19–1.14) | 0.153 |
| Vrinnevisjukhuset i Norrköping | 509 | 7 | 1.38 | 0.51 (0.22–1.00) | 0.077 |
| SkaS Skövde | 221 | 3 | 1.36 |  |  |
| Universitetssjukhuset i Linköping | 320 | 4 | 1.25 | 0.46 (0.14–1.09) | 0.128 |
| Falu lasarett | 502 | 6 | 1.20 | 0.44 (0.17–0.90) | 0.047 |
| Östersunds Sjukhus | 171 | 2 | 1.17 |  |  |
| Skellefteå lasarett | 173 | 2 | 1.16 |  |  |
| Blekingesjukhuset Karlskrona | 179 | 2 | 1.12 |  |  |
| Västerviks sjukhus | 198 | 1 | 0.51 |  |  |
| Akademiska Sjukhuset | 392 | 1 | 0.26 |  |  |
| Lycksele lasarett | 47 | 0 | 0.00 |  |  |
| Mora lasarett | 146 | 0 | 0.00 |  |  |

JF/JG KVÅ codes (n ≥ 40): NPOU incidence per code and univariable OR (code vs all other operations)

| KVÅ code | Title | Operations, n | NPOU, n | NPOU, % | OR (95% CI) | p |
| --- | --- | --- | --- | --- | --- | --- |
| JFH10 | Colectomy with ileostomy | 101 | 9 | 8.9 | 3.69 (1.72–6.98) | <0.001 |
| JFF11 | Laparoscopic loop ileostomy | 260 | 22 | 8.5 | 3.58 (2.22–5.48) | <0.001 |
| JFF13 | Enterostomy | 71 | 5 | 7.0 | 2.83 (0.99–6.40) | 0.026 |
| JFF27 | Laparoscopic sigmoidostomy | 247 | 17 | 6.9 | 2.82 (1.64–4.53) | <0.001 |
| JFF26 | Sigmoidostomy | 129 | 8 | 6.2 | 2.48 (1.11–4.79) | 0.014 |
| JFB60 | Sigmoid colon resection with sigmoidostomy | 160 | 9 | 5.6 | 2.24 (1.05–4.17) | 0.020 |
| JGB30 | Abdominoperineal resection (APR) | 335 | 18 | 5.4 | 2.16 (1.28–3.41) | 0.002 |
| JFB20 | Ileocecal resection | 245 | 13 | 5.3 | 2.12 (1.14–3.59) | 0.010 |
| JFB53 | Sigmoid colon resection with concomitant partial rectal resection | 108 | 5 | 4.6 | 1.81 (0.64–4.03) | 0.198 |
| JGB10 | Rectal resection with sigmoidostomy and closure of the rectal stump (Hartmann’s procedure) | 198 | 9 | 4.5 | 1.78 (0.84–3.31) | 0.094 |
| JGB31 | Laparoscopic and perineal abdominoperineal resection | 661 | 29 | 4.4 | 1.76 (1.17–2.55) | 0.004 |
| JFB00 | Small bowel resection | 572 | 24 | 4.2 | 1.67 (1.06–2.49) | 0.018 |
| JFF23 | Transverse colostomy | 48 | 2 | 4.2 | 1.61 (0.26–5.24) | 0.508 |
| JFG10 | Closure of loop colostomy without bowel resection | 49 | 2 | 4.1 | 1.58 (0.26–5.12) | 0.527 |
| JFB61 | Laparoscopic sigmoid colon resection with sigmoidostomy and rectal stump closure | 99 | 4 | 4.0 | 1.57 (0.48–3.77) | 0.382 |
| JGB34 | Laparoscopy-assisted abdominoperineal resection with intersphincteric dissection | 125 | 5 | 4.0 | 1.55 (0.55–3.44) | 0.339 |
| JGB00 | Rectal resection with total mesorectal excision (TME) | 280 | 10 | 3.6 | 1.38 (0.68–2.48) | 0.321 |
| JFB33 | Other resection involving small bowel and colon | 85 | 3 | 3.5 | 1.36 (0.33–3.65) | 0.604 |
| JFH00 | Colectomy with ileorectal anastomosis | 90 | 3 | 3.3 | 1.28 (0.31–3.43) | 0.675 |
| JFF10 | Loop ileostomy | 231 | 7 | 3.0 | 1.16 (0.49–2.30) | 0.700 |
| JFB46 | Sigmoid colon resection | 873 | 26 | 3.0 | 1.15 (0.75–1.69) | 0.504 |
| JGB01 | Laparoscopic rectal resection with total mesorectal excision (TME) | 646 | 19 | 2.9 | 1.13 (0.68–1.75) | 0.610 |
| JFK10 | Adhesiolysis | 148 | 4 | 2.7 | 1.03 (0.32–2.45) | 0.954 |
| JFB44 | Laparoscopic left hemicolectomy | 428 | 11 | 2.6 | 0.98 (0.50–1.71) | 0.940 |
| JFH20 | Proctocolectomy with ileostomy | 40 | 1 | 2.5 | 0.95 (0.05–4.39) | 0.960 |
| JFB30 | Right hemicolectomy | 1,897 | 47 | 2.5 | 0.93 (0.68–1.26) | 0.661 |
| JFB43 | Left hemicolectomy | 470 | 11 | 2.3 | 0.88 (0.45–1.54) | 0.692 |
| JFH11 | Laparoscopic colectomy with ileostomy | 185 | 4 | 2.2 | 0.82 (0.25–1.94) | 0.691 |
| JFH01 | Laparoscopic colectomy with ileorectal anastomosis | 93 | 2 | 2.2 | 0.81 (0.13–2.58) | 0.773 |
| JFB31 | Laparoscopic right hemicolectomy | 3,224 | 71 | 2.2 | 0.80 (0.61–1.03) | 0.087 |
| JFB01 | Laparoscopic small bowel resection | 101 | 2 | 2.0 | 0.75 (0.12–2.37) | 0.684 |
| JGB96 | Other rectal resection or extirpation | 51 | 1 | 2.0 | 0.74 (0.04–3.39) | 0.766 |
| JFB54 | Laparoscopic sigmoid colon resection with concomitant partial rectal resection | 207 | 4 | 1.9 | 0.73 (0.22–1.72) | 0.530 |
| JFB40 | Transverse colon resection | 104 | 2 | 1.9 | 0.73 (0.12–2.30) | 0.653 |
| JGB03 | Rectal resection with partial mesorectal excision | 105 | 2 | 1.9 | 0.72 (0.12–2.27) | 0.644 |
| JGB11 | Laparoscopic Hartmann’s procedure | 264 | 5 | 1.9 | 0.71 (0.25–1.56) | 0.454 |
| JGB33 | Abdominoperineal resection with intersphincteric dissection | 110 | 2 | 1.8 | 0.68 (0.11–2.17) | 0.596 |
| JFG26 | Closure of enterostomy with anastomosis to colon | 57 | 1 | 1.8 | 0.66 (0.04–3.01) | 0.682 |
| JFB47 | Laparoscopic sigmoid colon resection | 1,590 | 28 | 1.8 | 0.64 (0.42–0.92) | 0.023 |
| JFG00 | Closure of loop enterostomy without bowel resection | 243 | 4 | 1.6 | 0.62 (0.19–1.46) | 0.339 |
| JGA75 | Transanal endoscopic microsurgery (TEM) excision of rectal lesion | 396 | 6 | 1.5 | 0.56 (0.22–1.16) | 0.166 |
| JGB04 | Laparoscopic rectal resection with partial mesorectal excision | 276 | 4 | 1.4 | 0.54 (0.17–1.28) | 0.224 |
| JFG36 | Closure of colostomy with anastomosis to rectum | 141 | 2 | 1.4 | 0.53 (0.09–1.67) | 0.375 |
| JFB21 | Laparoscopic ileocecal resection | 308 | 4 | 1.3 | 0.48 (0.15–1.14) | 0.149 |
| JFB50 | Other colon resection | 80 | 1 | 1.2 | 0.47 (0.03–2.11) | 0.450 |
| JFG40 | Revision of entero-/colostomy without laparotomy | 86 | 1 | 1.2 | 0.43 (0.02–1.96) | 0.407 |
| JFG20 | Closure of enterostomy with bowel resection | 94 | 1 | 1.1 | 0.40 (0.02–1.79) | 0.358 |
| JGC01 | Laparoscopic rectopexy | 297 | 2 | 0.7 | 0.25 (0.04–0.77) | 0.049 |
| JGA73 | Transanal excision of rectal lesion | 161 | 1 | 0.6 | 0.23 (0.01–1.03) | 0.143 |
| JFF30 | Other colostomy | 42 | 0 | 0.0 | 0.00 (0.00–0.13) | 0.958 |
